# Anti-Inflammatory Effects of *Heritiera littoralis* Fruits on Dextran Sulfate Sodium- (DSS-) Induced Ulcerative Colitis in Mice by Regulating Gut Microbiota and Suppressing NF-*κ*B Pathway

**DOI:** 10.1155/2020/8893621

**Published:** 2020-12-05

**Authors:** Guosheng Lin, Minyao Li, Nan Xu, Xiaoli Wu, Jingjing Liu, Yulin Wu, Qian Zhang, Jian Cai, Changjun Gao, Ziren Su

**Affiliations:** ^1^Research Center of Chinese Herbal Resource Science and Engineering, Key Laboratory of Chinese Medicinal Resource from Lingnan, Ministry of Education, Guangzhou University of Chinese Medicine, Guangzhou 510006, China; ^2^School of Pharmaceutical Science (Mathematical Engineering Academy of Chinese Medicine), Guangzhou University of Chinese Medicine, Guangzhou 510006, China; ^3^School of Biomedical and Pharmaceutical Sciences, Guangdong University of Technology, Guangzhou 510006, China; ^4^Guangdong Academy of Forestry, Guangzhou 510520, China; ^5^Guangdong Provincial Key Laboratory of Silviculture, Protection and Utilization, Guangzhou 510520, China

## Abstract

**Materials and Methods:**

The chemical compositions of EFH were identified using LC-ESI-MS. The mice with 3% DSS-induced UC were administered EFH (200, 400, and 800 mg/kg), sulfasalazine (SASP, 200 mg/kg), and azathioprine (AZA, 13 mg/kg) for 10 days via daily gavage. The colonic inflammation was evaluated by the disease activity index (DAI), colonic length, histological scores, and levels of inflammatory mediators. The gut microbiota was characterized by 16S rRNA gene sequencing and analysis.

**Results:**

LC-ESI-MS analysis showed that EFH was rich in alkaloids and flavones. The results indicated that EFH significantly improved the DAI score, relieved colon shortening, and repaired pathological colonic variations in colitis. In addition, proteins in the NF-*κ*B pathway were significantly inhibited by EFH. Furthermore, EFH recovered the diversity and balance of the gut microbiota.

**Conclusions:**

EFH has protective effects against DSS-induced colitis by keeping the balance of the gut microbiota and suppressing the NF-*κ*B pathway.

## 1. Introduction

Ulcerative colitis (UC) belongs to the inflammatory bowel disease (IBD) family and is a nonspecific chronic inflammatory disease (Do et al., 2020). UC clinically appears as chronic diarrhea, mucous-filled and bloody purulent stools, abdominal pain, weight loss, and fatigue [1], which all seriously affect people's quality of life. The current treatments for UC are aminosalicylic drugs, glucocorticoid drugs, immunosuppressants, and monoclonal antibodies, but these drugs cannot achieve ideal results because of the unclear etiology of UC [2]. As the morbidity of UC and the incidence of disease flares worsen, people increasingly turn to alternative medicine approaches [3], so there is a growing need to develop novel, efficient, and safe candidates for UC treatment.

*Heritiera littoralis* Dryand. (Sterculiaceae), a semimangrove plant, is typically found in mangrove zones, which are mostly tropical and subtropical areas [4]. Mangroves not only protect the environment but also have medicinal value. In terms of medicinal uses, *Heritiera littoralis* Dryand. has been used to treat indigestion, diarrhea, and dysentery; the medicinal parts are mainly in the seeds and are obtained by decoction [5]. Existing literature had reported that the *Heritiera littoralis* bark exerted its anti-inflammatory effects by reducing the release of NO, PGE2, and TNF-*α*, as well as by downregulating iNOS and COX-2 [6]. In addition, *Heritiera littoralis* leaf extracts have exhibited obvious antibacterial and anticancer activities [7, 8]. The pharmacodynamics of the *Heritiera littoralis* fruit has not been clearly reported in publications, but the fruit does contain anti-inflammatory chemicals, such as flavonoids and triterpenoids [9]. Therefore, we hypothesized that the extracts of the *Heritiera littoralis* fruit (EFH) may have anti-inflammatory effects.

In this study, we proposed that UC is caused by the imbalance of intestinal microbiota, associated with intestinal inflammation responses [10]. NF-*κ*B contributes to the mechanism of the inflammatory process and controls the release of inflammatory cytokines involved in UC [11]. Therefore, we hypothesized that gut microbiota dysbiosis would improve after introduction of EFH to the intestinal microenvironment, as a result of changes to the release of inflammatory cytokines and inhibition of the NF-*κ*B pathway. In our research, we investigated the mechanism of action for EFH in dextran sulfate sodium- (DSS-) induced colitis in an experimental murine model.

## 2. Materials and Methods

### 2.1. Materials

The *Heritiera littoralis* fruits were provided by the Guangdong Academy of Forestry and identified by Professor Jian Cai of that academy. The voucher specimens (914556) were deposited for further reference in South China Botanical Garden (Guangzhou, China). Dextran sulfate sodium was bought from MP Biomedicals (Montreal, Canada). Sulfasalazine (SASP) was provided by Shanghai Xinyi Tianping Pharmaceutical Co., Ltd. (Shanghai, China) and azathioprine (AZA) by Aspen Pharmacare Australia Co., Ltd, (Australia). The ELISA kits for TNF-*α*, IFN-*γ*, IL-1*β*, and IL-6 were bought from the Shanghai MLBIO Biotechnology Co., Ltd. (Shanghai, China). The kit for biochemical analysis of myeloperoxidase (MPO) was obtained from the Nanjing Jiancheng Bioengineering Institute (Nanjing, Jiangsu, China). The antibodies (NF-*κ*B p65, NF-*κ*B p-p65, I*κ*B*α*, p-I*κ*B*α*, IKK*α*, p-IKK*α*, *β*-actin, and Histone H3) were purchased from Affinity Biosciences (Ohio, USA). All other reagents and chemicals were of analytical grade.

### 2.2. Preparation of Extracts

The *Heritiera littoralis* fruits were collected from the Nansha Wetland Park (Guangdong, Guangzhou, China). The *Heritiera littoralis* fruits were dried at 60°C, pulverized and filtered through an 80-mesh sieve to obtain a dry product for use. The dried fruits (100 g) were extracted by 20-fold distilled water for 2 h. After filtration, the residue was reextracted by 20-fold distilled water under the same condition. Thereafter, the two filtrates were combined in a container and then evaporated to 100 mL. Finally, the fruit extracts were stored in a refrigerator at a concentration of 1 g/mL for subsequent animal experiments [12, 13].

### 2.3. LC-ESI-MS Analysis

The extracts of EFH for chemical composition analysis were precipitated by 95% ethanol. The filtrate was concentrated to dryness in vacuum and dissolved in methanol. The EFH was analyzed by liquid chromatography-electrospray ionization mass spectrometry (LC-ESI-MS) with ESI-MS-positive and ESI-MS-negative ion acquisition modes. The sample (5 *μ*L) was injected into the UPLC apparatus equipped with a reverse phase C-18 column (150 × 2.1 mm 1.8 *μ*m, Welch). Mobile phase elution was performed with a flow rate of 0.3 mL/min using water acidified with 0.1% formic acid (A) and acetonitrile acidified with 0.1% formic acid (B), as follows: 0-1 min, 98-98% A (*v*/*v*), 2-2% B (*v*/*v*); 1-5 min, 98-80% A, 2-20% B; 5-10 min, 80-50% A, 20-50% B; 10-15 min, 50-20% A, 50-80% B; 15-20 min, 20-5% A, 80-95% B; 20-25 min, 5-5% A, 95-95% B; and 25-26 min, 5-98% A, 95-2% B; and 26-30 min, 98-98% A, 2-2% B. The positive ion mode was adjusted to a 300°C capillary temperature and 3 kV capillary voltage. All data collected were acquired and processed by the MassLynx 4.1 software.

### 2.4. Experimental Animals

The animals (male BALB/c mice, 22-25 g) were bought from the Guangzhou University of Traditional Chinese Medicine (SYXK (YUE) 2018-0034, Guangzhou, China). All animal housing (23 ± 2°C, 50 ± 5% humidity, 12 h light and dark cycle), handling, and feeding were supervised by the Animal Experimentation Ethics Committee at Guangzhou University of Traditional Chinese Medicine (Registration no. 20181224002).

After 7-day acclimation, all animals were assigned into 7 groups (*n* = 12) and received their respective treatments by oral gavage, as follows: control group and DSS group (distilled water, 0.1 mL/10 g), SASP-supplemented group (200 mg/kg), AZA-supplemented group (13 mg/kg), and EFH-supplemented groups (200, 400, and 800 mg/kg, respectively). During the experiment, all groups except for the control group were exposed to 3% DSS drinking freely for 10 days [14], and relative oral treatments were carried out once daily. The consumption of distilled water versus DSS solution between the groups was similar. The administrated doses of treatments were selected according to results from our pilot study and previous publications [15]. On the last day of the experiment, all the mice were sacrificed by carbon dioxide euthanasia; then, the colons were quickly removed and stored at −80°C for further analysis.

### 2.5. Evaluation of Disease Activity Index (DAI)

The DAI (body weight loss, stool character, and bloody feces) was evaluated by an observer daily, and the DAI score (calculated as [weight loss score + fecal trait score + hematochezia score]/3) was regarded as the standard scoring system [16]. After the mice were sacrificed, the colorectal lengths were measured. Portions of the distal colorectums were embedded and stained with hematoxylin and eosin (H&E). The histological scores were evaluated in a blinded manner [17].

### 2.6. Evaluation of the Levels of TNF-*α*, IFN-*γ*, IL-1*β*, IL-6, and MPO

The colorectal tissues were homogenized, and supernatants were collected by centrifugation. The levels of TNF-*α*, IFN-*γ*, IL-1*β*, and IL-6 were measured using ELISA kits at 450 nm according to the manufacturer's protocols, whereas MPO activity was determined with a myeloperoxidase assay kit at 460 nm.

### 2.7. Quantitative Real-Time PCR Analysis

The total RNA was extracted from the colonic tissue with TRIzol reagent. After RNA concentration and purity control were completed, the high-quality RNA samples (RNA concentration ≥ 500 ng/*μ*L, OD260/OD280 ≥ 2.0) were reverse transcribed to complementary DNA (cDNA) using FastKing Reverse Transcriptase Kit according to instructions from the supplier. The sequences of the primers used in this study are listed in Table 1. The QRT-PCR was performed with a ChamQ SYBR qPCR Master Mix (Vazyme Biotech Co., Ltd., Nanjing, China) and CFX Manager software (Bio-Rad Laboratories Inc.). The protocol was 95°C for 10 min, followed by 95°C for 15 s and 60°C for 20 s; this sequence was repeated for 40 cycles. The mRNA expression level (iNOS, COX-2, IL-4, IL-12, and IL-17) was calculated with the 2^-*ΔΔ*Ct^ method relative to control gene GAPDH.

### 2.8. Western Blot Analysis

The total, cytoplasmic, and nuclear proteins were extracted using their corresponding extraction kits according to the manufacturer's statements. Briefly, the colonic tissues were homogenized with RIPA lysis buffer on ice. After incubation (20 min) and centrifugation (14000 × g, 4°C, 10 min), the supernatant was collected as total protein for further analysis. The other samples were homogenized with PBS and centrifuged for 3 min (500 × g, 4°C). The sediment was mixed with Buffer A thoroughly for 30 min and then centrifuged (12000 × g, 4°C, 10 min) again. The cytoplasmic protein was separated from the supernatant, whereas the nuclear protein was extracted from the sediment with Buffer B. After separated by SDS-PAGE, the proteins above were transferred onto PVDF membranes. Subsequently, the membranes were blocked in 5% nonfat-dried milk and incubated with primary antibodies NF-*κ*B p-p65, NF-*κ*B p65, I*κ*B*α*, p-I*κ*B*α*, IKK*α*, and p-IKK*α* (all 1 : 1000 dilution) overnight, followed by incubation with secondary antibody. The protein density was quantified by ImageJ software relative to *β*-actin or Histone H3.

### 2.9. Gut Microbiota 16S rRNA Gene Sequencing and Analysis

Fecal genomic DNA was extracted using the DNA kit. The 16S rDNA V4 region of the rRNA gene was amplified by PCR with the primer (515F: 5′-GTGCCAGCMGCCGCGGTAA-3′ and 806R: 5′-GGACTACHVGGGTATCTAAT-3′). Purified amplicons were sequence paired on an Illumina platform. The effective tags were clustered into operational taxonomic units (OTUs) of ≥97% similarity using USEARCH (v7.0.1090). On the basis of the relative abundance of OTUs, the microbial diversity and structural classification were further analyzed using programming language R (v3.1.1).

### 2.10. Statistical Analysis

All data were presented as the mean ± standard error of mean (SEM) with Statistical Product and Service Solutions (SPSS) software (version 23.0). Statistical analysis was carried out by one-way ANOVA followed by LSD test and Dunnett's test. A value of *p* < 0.05 was considered statistically significant.

## 3. Results

### 3.1. The Phytochemical Constituent Analysis of EFH

**Figure 1:**
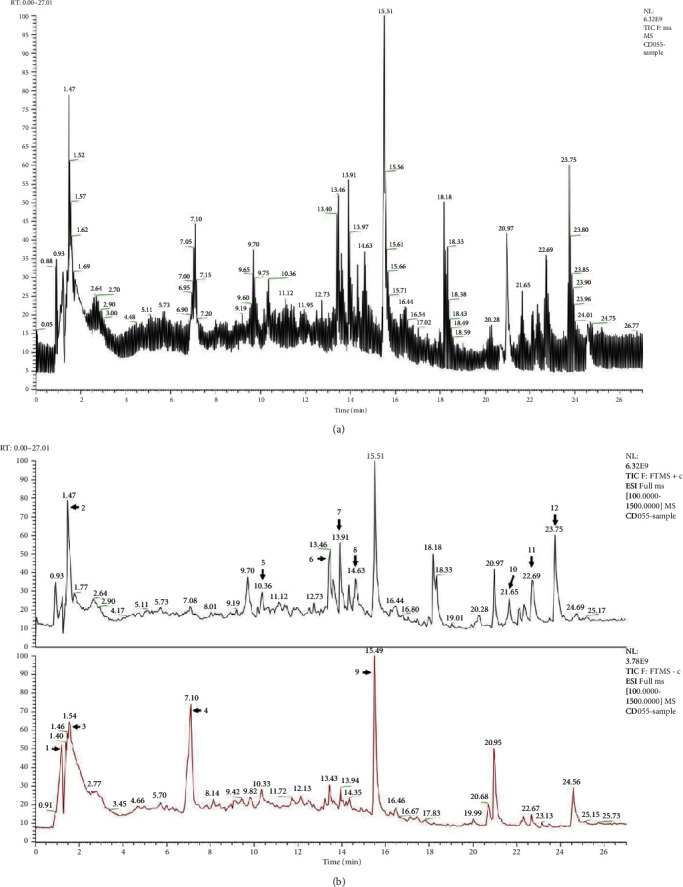
The LC-ESI-MS chromatograms of EFH. (a) The total ion currents of EFH. (b) Positive mode in black and negative mode in red. Peak assignments are listed in Table 2.

The LC-ESI-MS analysis revealed that the high constituents were alkaloids and flavones in EFH. As shown in Figure 1 and Table 2, the chromatogram identified the phytochemical constituents in EFH as L-glutamic acid, triglycidyl glycerol, 2-naphthalenesulfonic acid, penicillic acid, syringic acid, puerarin, taxifolin, 4-hydroxycoumarin, carbidopa, N,N-dimethylsphingosine, caffeic acid, and 7-methylxanthine. These findings were consistent with previous publications.

### 3.2. EFH Ameliorated Inflammatory Indices of DSS-Induced Colitis

**Figure 2:**
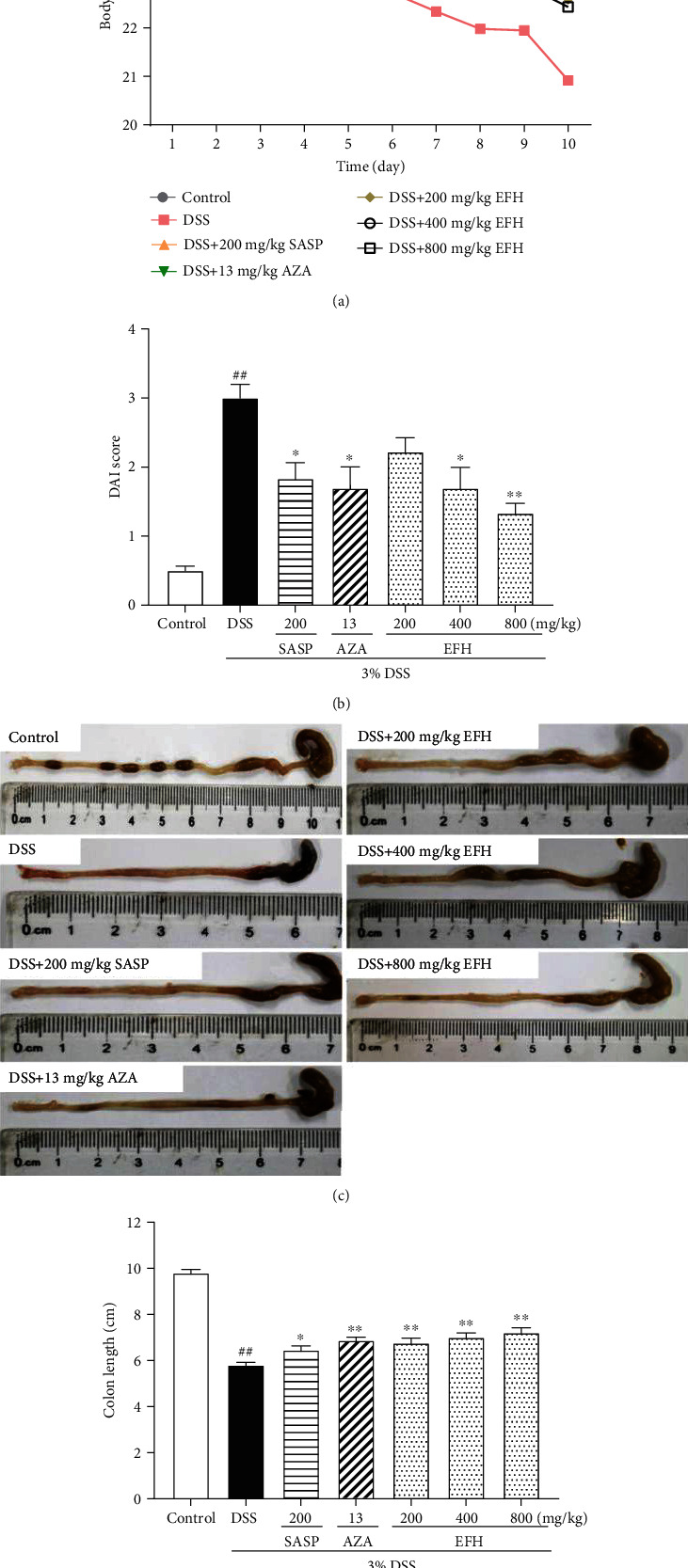
EFH ameliorated inflammatory indices of DSS-induced colitis. (a) The daily body weight changes from 1^st^ day to 10^th^ day. (b) The disease activity index (DAI) in mice. (c) The length of colons from a macroscopic perspective. (d) Quantitative measurement of colon length. Data are presented as the means ± S.E.M. (*n* = 12). ^**##**^*p* < 0.01 vs. the control group; ∗*p* < 0.05, ∗∗*p* < 0.01 vs. the DSS group.

The mice treated freely with 3% DSS were used successfully to establish colitis. As shown in Figure 2(a), the body weight decrease on day 4 was significantly greater in the DSS group compared to the control group. However, the mice in the EFH groups exhibited a slight decrease in weight loss especially from day 5. The results indicated that EFH treatment effectively alleviated DSS-induced weight loss.

Figure 2(b) showed that the DAI score of the DSS group dramatically increased (*p* < 0.01), whereas EFH treatment dose dependently decreased DAI scores even more than treatment with SASP or AZA. In addition, the DAI score was inversely associated with colon length, which was consistent with previous publication [29]. In Figures 2(c) and 2(d), the colon length in the DSS group was the shortest (*p* < 0.01), which was reversed by EFH in a dose-dependent manner (all *p* < 0.01).

### 3.3. EFH Suppressed Inflammatory Infiltration in DSS-Induced Colitis

**Figure 3:**
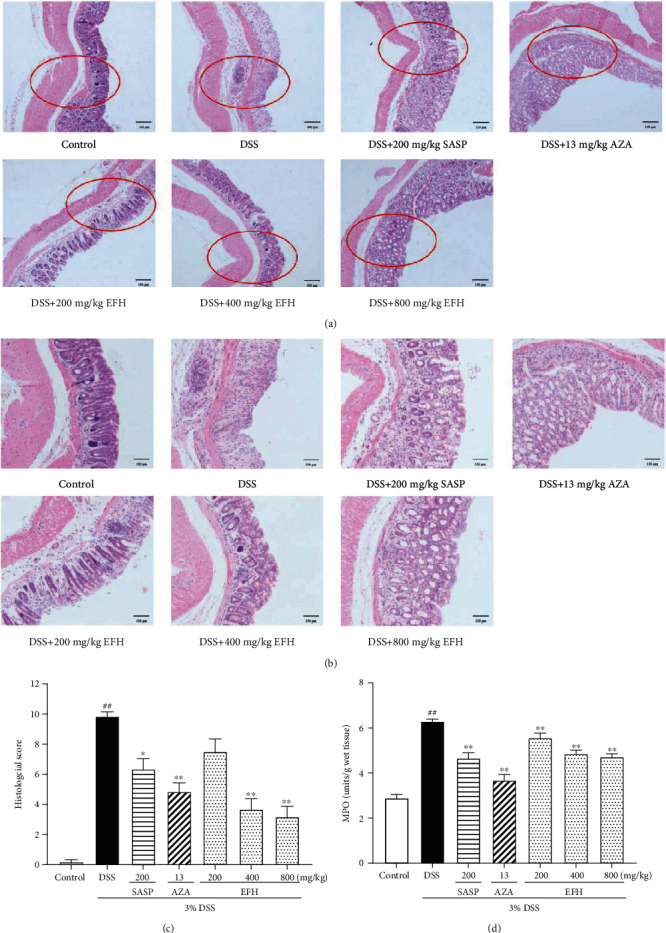
EFH suppressed inflammatory infiltration in DSS-induced colitis. H&E staining of the formalin-fixed sections of colon (scale bars, 100 *μ*m). (a) Magnification ×200 (the parts in red circles were enlarged), (b) magnification ×400, (c) histological scores, and (d) MPO activity of colon tissue in DSS-induced colitis mice. Data are presented as the means ± S.E.M. (*n* = 10). ^**##**^*p* < 0.01 vs. the control group; ∗*p* < 0.05, ∗∗*p* < 0.01 vs. the DSS group.

The results of H&E staining and histopathological scores showed that the colonic tissue structures in the DSS group were destroyed compared to the control group (Figures 3(a) and 3(b)). Conversely, the groups treated with EFH reversed the changes in gut epithelial general morphology (*p* < 0.01). In addition, the MPO activity in DSS-treated mice remarkably increased compared to the control group (*p* < 0.01), suggesting growing infiltration of inflammatory cells. However, the groups treated with EFH, SASP, and AZA resulted in suppressing the MPO activity. Therefore, our results indicated that EFH significantly protected the intestinal epithelial structure.

### 3.4. EFH Suppressed the Levels of TNF-*α*, IFN-*γ*, IL-1*β*, and IL-6

**Figure 4:**
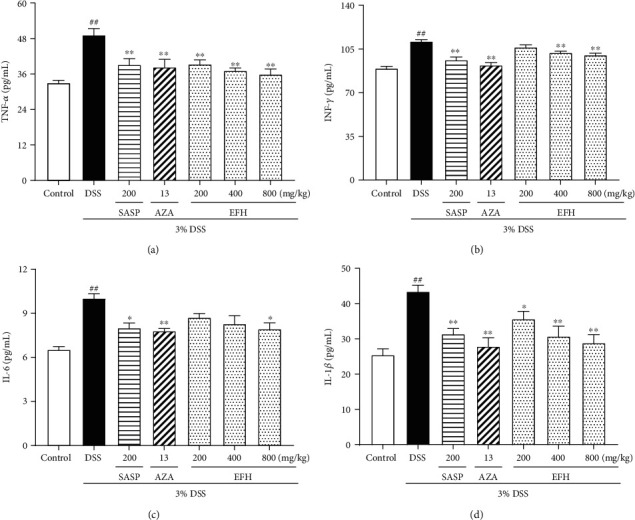
Effects of EFH on colonic level of proinflammatory cytokines TNF-*α* (a), IFN-*γ* (b), IL-1*β* (c), and IL-6 (d) as determined by ELISA in DSS-induced colitis mice. Data are presented as the means ± S.E.M. (*n* = 8). ^**##**^*p* < 0.01 vs. the control group; ∗*p* < 0.05, ∗∗*p* < 0.01 vs. the DSS group.

To evaluate the anti-inflammatory effects of EFH, we measured the activity of proinflammatory cytokines by ELISA kits. As showed in Figure 4, the levels of all proinflammatory cytokines in the DSS group were much higher than those in the control group (all *p* < 0.01). EFH treatment markedly lowered the expressions of TNF-*α*, IFN-*γ*, IL-1*β*, and IL-6, and the difference was significant in the mice treated with 800 mg/kg of EFH. Furthermore, the decrease observed in the mice receiving 800 mg/kg of EFH was close to that of the SASP and AZA groups.

### 3.5. EFH Decreased the mRNA Expressions of COX-2, iNOS, IL-12, IL-17, and IL-4

**Figure 5:**
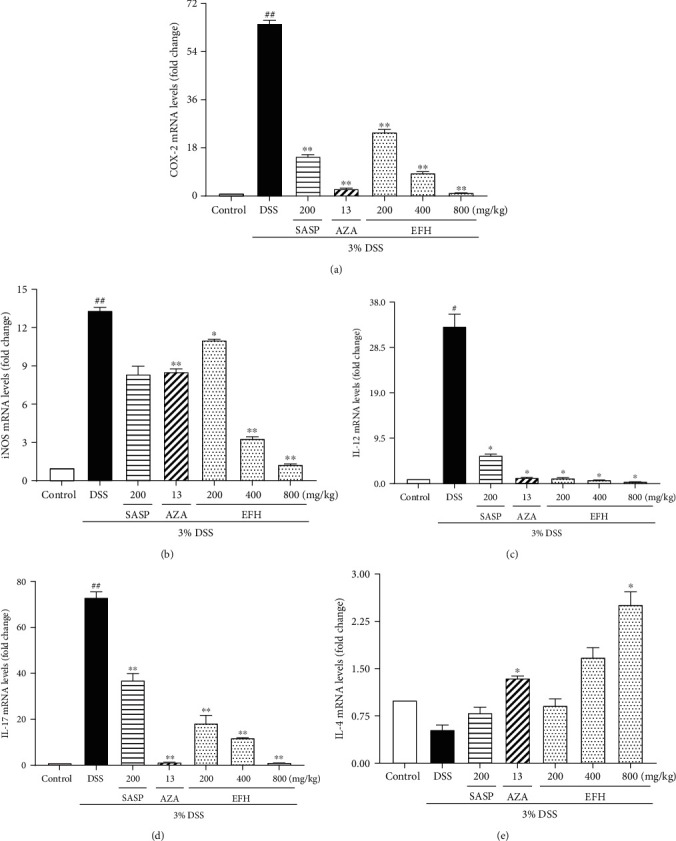
Effects of EFH on mRNA expression levels of COX-2 (a), iNOS (b), IL-12 (c), IL-17 (d), and IL-4 (e) in colorectums as determined by q-PCR in DSS-induced colitis mice. Data are presented as the means ± S.E.M. (*n* = 3). ^**#**^*p* < 0.05, ^**##**^*p* < 0.01 vs. the control group; ∗*p* < 0.05, ∗∗*p* < 0.01 vs. the DSS group.

Many proinflammatory cytokines (COX-2, iNOS, IL-12, and IL-17) and an anti-inflammatory cytokine (IL-4) are involved in the initiation and development of intestinal inflammation. As shown in Figure 5, the levels of COX-2, iNOS, IL-12, and IL-17 in the DSS group were significantly higher than the level in the control group, and these high levels were reversed by EFH, SASP, or AZA. However, the anti-inflammatory cytokine IL-4 was upregulated in the EFH groups but was downregulated in the DSS group.

### 3.6. EFH Inhibited the Expressions of NF-*κ*B Pathway

**Figure 6:**
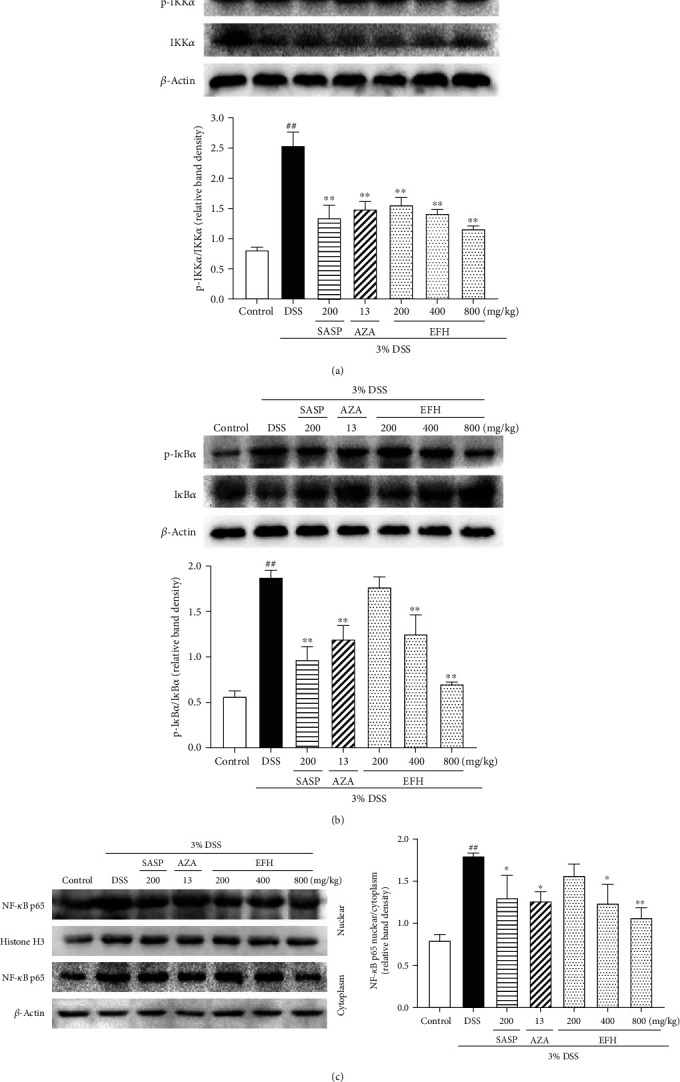
Effects of EFH on colonic expressions of the NF-*κ*B pathway proteins determined by western blot in DSS-induced colitis mice. Densitometry analyses of western blots were determined by quantifying the protein levels of p-IKK*α*/IKK*α* (a), p-I*κ*B*α*/I*κ*B*α* (b), and NF-*κ*B p65 nuclear/cytoplasm (c). Data are presented as the means ± S.E.M. (*n* = 3). ^**##**^*p* < 0.01 vs. the control group; ∗*p* < 0.05, ∗∗*p* < 0.01 vs. the DSS group.

The NF-*κ*B pathway plays an important role in the intestinal inflammation reaction, which is activated by I*κ*B*α* and IKK*α* phosphorylation [30]. The colonic tissue cell in the DSS group translocated NF-*κ*B p-p65 to the nucleus by phosphorylating IKK*α* and I*κ*B*α* [31]. As shown in Figure 6, the activated NF-*κ*B p65 (*p* < 0.05) and the phosphorylation of IKK*α* and I*κ*B*α* (all *p* < 0.01) in the DSS group were significantly higher than in the control group. On the contrary, treatment with EFH downregulated the expressions of NF-*κ*B p-p65 p-IKK*α*, and p-I*κ*B*α*, and these changes were similar to the effects of SASP and AZA.

### 3.7. EFH Altered Microbial Structure and Diversity of Gut Microbiota in Mice

**Figure 7:**
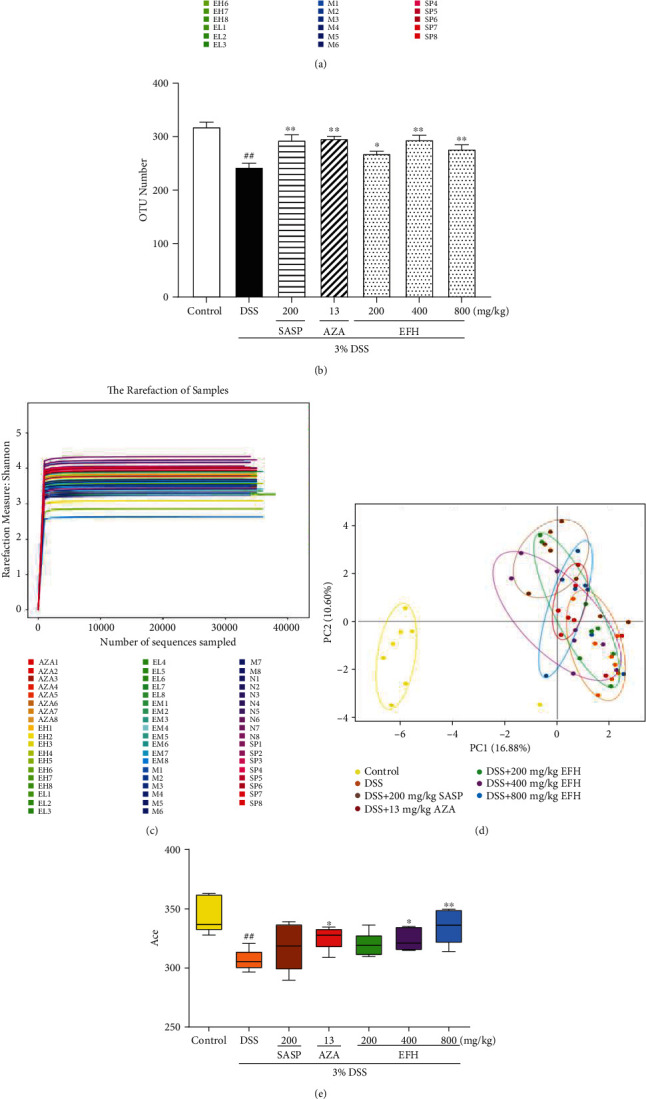
Effects of EFH on the overall structure and microbial diversity of the gut microbiota in DSS-induced colitis mice. (a) OTU rank curve. (b) The histogram of OTU number. (c) Shannon's rarefaction curve of samples. (d) Multiple sample PCA analysis. (e) Ace and (f) Chao of alpha diversity analysis. Data are presented as the means ± S.E.M. (*n* = 3). ^**##**^*p* < 0.01 vs. the control group; ∗*p* < 0.05, ∗∗*p* < 0.01 vs. the DSS group.

**Figure 8:**
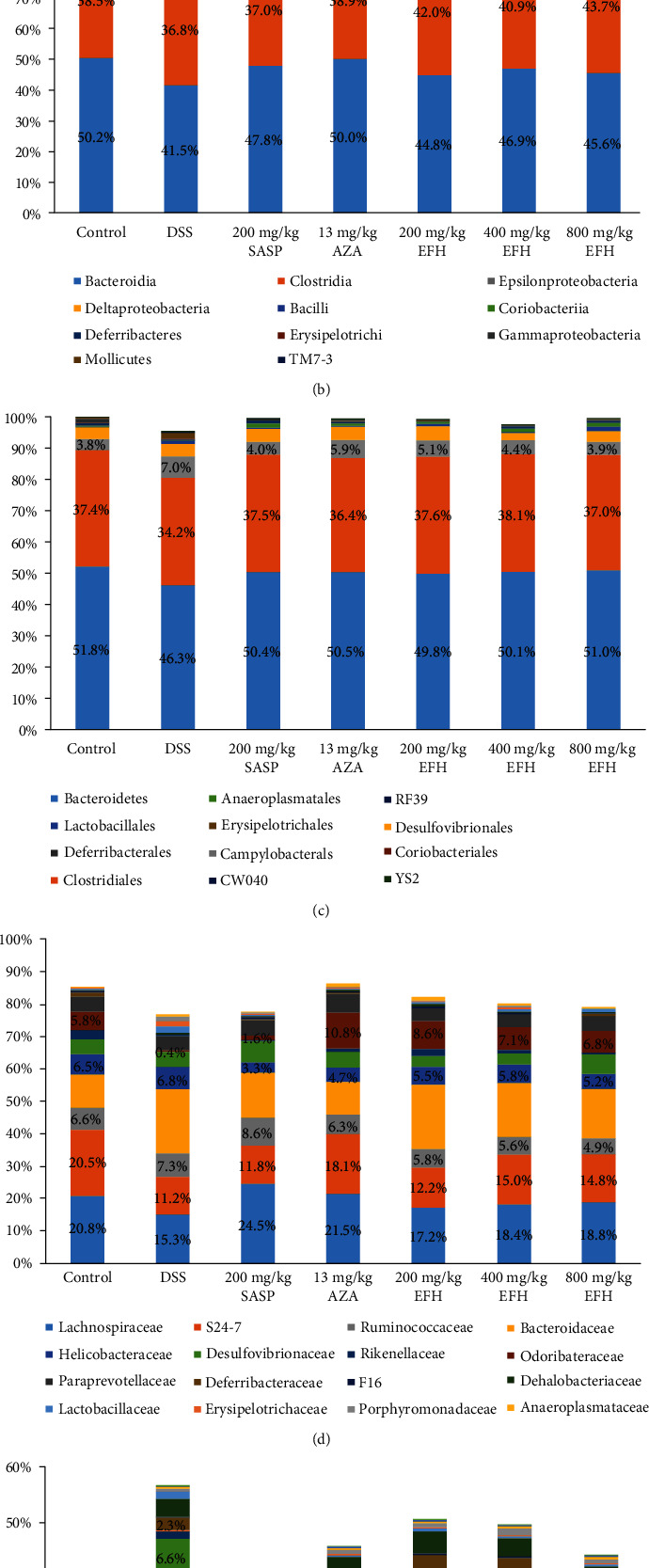
Effects of HQD on structural segregation of the gut microbiota in DSS-induced colitis mice. (a) Phylum, (b) class, (c) order, (d) family, and (e) genus (*n* = 5).

Changes in the intestinal microenvironment have been reported in UC [32]. The operational taxonomic units (OTU) rank curve represents the number of observed OTUs in all samples. As showed in Figure 7(b), the DSS group had significantly fewer OTUs than other groups (*p* < 0.01), but the number of OTUs was enriched by EFH treatment. Additionally, the plateaued rarefaction curve in Figure 7(c) indicated that the sequencing depth of all samples had basically finished. The principal component analysis (PCA) revealed that the microbial community structure of the DSS group deviated from that of control group, whereas EFH treatment partially mitigated the shift (Figure 7(d)). According to alpha diversity analysis (Figures 8(e) and 8(f)), a significant reduction in microbial diversity was observed in the DSS group, but higher diversity was recorded in the EFH groups.

### 3.8. EFH Regulated Gut Microbiota Structure in DSS-Induced Colitis Mice

The histograms in Figure 8 revealed the gut microbiota community structure and the marked percentage showed the relative abundance at all levels. The results indicated that DSS decreased the relative abundance of Bacteroidetes and Firmicutes by 2.4% and 3.2%, respectively, compared with the control group, whereas DSS increased the levels of Proteobacteria by 6.8%. However, EFH reversed the proportions of all 3 phyla to beyond those of the control group. At the class level (Figure 8(b)), the levels of *Bacteroidia* and *Clostridia* dropped in the DSS group, which was reversed by EFH treatment. In addition, *ɛ-Proteobacteria* and *δ-proteobacteria* related to IBD pathogenesis [33] were found at high levels in the DSS group.

As shown in Figure 8(c), *Bacteroidales*, *Clostridiales*, *Campylobacterales*, and *Desulfovibrionales* were the most represented of the 12 bacterial orders. Among them, *Campylobacterales* was identified at a relatively high level in the DSS group but was detected only slightly in the EFH groups. Most of the 16 families observed were related to the communities observed at the phylum and class levels. *Lachnospiraceae* and *S24-7* were the main intestinal floras in the control group, and their levels were reduced by DSS treatment. After EFH treatment, the levels of these bacteria recovered to levels similar to the control group.

As shown in Figure 8(e), sequencing data identified 18 genera of intestinal microflora. The relative abundance of *Odoribacter* was significantly downregulated by DSS treatment, but EFH treatment reversed the alteration. In addition, EFH reduced the levels of *Helicobacter*, *Ruminococcus*, and *Paraprevotella*.

## 4. Discussion

In this investigation, we proved for the first time the effectiveness of EFH to treat DSS-induced colitis by improving the balance of the gut microbiota and inhibiting the NF-*κ*B pathway. DSS-induced colitis is similar to UC in human [34], so we extrapolated the anti-UC mechanism of EFH based on the basis of results in mice with DSS-induced colitis.

Our LC-ESI-MS chromatogram results showed that EFH is a complex mixture of chemical compositions. The main effective components of EFH were flavonoids, predominantly taxifolin and puerarin. Taxifolin exerts its diverse therapeutic benefits in inflammation-related diseases via the inhibition of the NF-*κ*B pathway [35]. Puerarin is associated with changes in the gut microbiota and inhibits the NF-*κ*B pathway to relieve the inflammatory response [36]. Therefore, we speculated that the observed anti-inflammatory effects of EFH might be associated with flavonoids. However, more research is needed to clarify how the biologically active ingredients of EFH act on DSS-induced colitis.

The DAI score, colon length, and histological changes are some of the markers used to assess for inflammation in BALB/c mice with DSS-induced colitis [29]. Our results showed that mice treated with EFH maintained body weight and had significantly decreased DAI scores and histological scores. In addition, MPO activity was higher in the DSS group but was lowered with EFH treatment, suggesting that EFH attenuated DSS-induced massive inflammatory infiltration and disruption of mucosal structures [37]. These results suggested that EFH exerted noticeably protective effects against DSS-induced colitis to alleviate colonic inflammation.

In recent years, the gut microbiota has received great attention, and many studies have explored the changes of the intestinal flora occurring with intestinal diseases, including IBD and CRC [38]. In our study, DSS reduced the microbial diversity of the gut microbiota in mice with colitis [39]. In contrast, EFH improved the bacterial diversity and kept the balance between the population of beneficial bacteria and pathogenic bacteria. For example, *Helicobacter* is associated with chronic gastritis and peptic ulcer diseases [40], and we found that EFH reduced the relative increase of *Helicobacter* caused by DSS. Remarkably, EFH treatment increased the richness of *Odoribacter* compared with DSS treatment, and *Odoribacter* may reduce SCFA production to relieve UC [41]. These results also suggested that EFH regulated the gut microbiota to keep the balance of intestinal homeostasis in DSS-induced colitis. However, the relationship between the gut microbiota and colitis must be investigated further.

Dysbiosis of the gut microbiota is associated with the destruction of intestinal barrier and signals to epithelial cells to trigger inflammatory responses [42]. The dysbiosis of cytokines and gut bacteria might be controlled by the NF-*κ*B pathway [43]. The phosphorylation of p65 was thought to enhance its entrance into the nucleus, so we measured the expression level of the NF-*κ*B p65 nuclear/cytoplasm ratio. According to our results, EFH relieved DSS-induced inflammatory infiltration in epithelial cells and then suppressed the NF-*κ*B pathway through downregulation of the phosphorylated proteins IKK*α*, I*κ*B*α*, and NF-*κ*B p65. The activated NF-*κ*B pathway can be associated with the high expression of cytokines. Therefore, proinflammatory cytokines (TNF-*α*, IFN-*γ*, IL-1*β*, IL-6, IL-12, and IL-17) were expressed highly in the DSS group, but the anti-inflammatory cytokine (IL-4) expression was low, and these changes were reversed by EFH treatment. Proinflammatory cytokines mediate cell infiltration [44], whereas anti-inflammatory cytokines are involved in tissue growth, repair, and anti-inflammatory responses [45]. Therefore, EFH suppressed the NF-*κ*B pathway to upregulate the anti-inflammatory cytokine but downregulate proinflammatory cytokines, which formed an anti-inflammatory environment to improve colon tissue repair.

Activated NF-*κ*B transcriptionally expresses many proteins involved in the initiation of signal transduction cascades, especially COX-2 and iNOS [46]. In fact, iNOS works synergistically with COX-2 to release cytokines and contribute to the development of inflammatory reactions [47]. As expected, high expressions of iNOS and COX-2 in the DSS group were detected, and these were reduced with EFH treatment. The anti-inflammatory effects of EFH might not only reduce the release of inflammatory factors but also downregulate iNOS and COX-2 mRNA levels.

Furthermore, two approved drugs (SASP and AZA) were used to more comprehensively evaluate the efficacy of EFH. As shown in the graphical abstract, the two drugs are effective treatments for UC as measured clinically and pharmacologically [48]. SASP can decompose to 5-ASA to inhibit the synthesis and release of PGE2 (related to COX-2), and AZA can suppress the NF-*κ*B pathway through immune suppression. The results of this study, comparing EFH effects with those of SASP and AZA, suggest that EFH may be a potential better therapeutic agent than SASP or AZA for the treatment of UC.

## 5. Conclusions

Our study shows novel insights into the anti-inflammatory effects of EFH in DSS-induced colitis in mice. The protective effects of EFH may be associated with the regulation of the gut microbiota, suppression of the NF-*κ*B pathway, and subsequent downregulation of inflammatory mediators. Our investigation provides experimental evidence for the pharmaceutical application of EFH in the treatment of UC and encourages further study of this fruit.

## Figures and Tables

**Table 1:** Sequences of primers used for QRT-PCR.

Gene		Primer sequences (5′-3′)	Product size (bp)
iNOS	Reverse	CAGCCACATTGATCTCCGTGACAG	358
	Forward	GATGTGCTGCCTCTGGTCTTGC	
COX-2	Reverse	GCGGTTCTGATACTGGAACTGCTG	253
	Forward	TGGTCTGGTGCCTGGTCTGATG	
IL-17	Reverse	GGTCTTCATTGCGGTGGAGAGTC	222
	Forward	TGATGCTGTTGCTGCTGCTGAG	
IL-4	Reverse	CGAAAGAGTCTCTGCAGCTCCA	191
	Forward	GTCACAGGAGAAGGGACGCC	
IL-12	Reverse	GCAGACAGAGACGCCATTCCAC	378
	Forward	CACCTGTGACACGCCTGAAGAAG	
GAPDH	Reverse	TCGCTCCTGGAAGATGGTGATGG	235
	Forward	AATGGTGAAGGTCGGTGTGAACG	

**Table 2:** The phytochemical constituents of EFH.

Order	Retention time (R.t)	Components	Molecular weight	Area (%)	Ref.
1	1.397	L-glutamic acid	147.05313	4.34%	[18]
2	1.471	Triglycidyl glycerol	277.15236	8.97%	[19]
3	1.539	2-Naphthalenesulfonic acid	208.02116	10.82%	[20]
4	7.116	Penicillic acid	170.058	10.56%	[21]
5	10.361	Syringic acid	198.05271	0.92%	[22]
6	13.453	Puerarin	416.10993	1.99%	[23]
7	13.92	Taxifolin	304.05788	2.34%	[24]
8	14.621	4-Hydroxycoumarin	162.03058	1.32%	[22]
9	15.508	Carbidopa	226.09909	11.53%	[25]
10	22.645	N,N-Dimethylsphingosine	309.30278	1.16%	[26]
11	22.696	Caffeic acid	180.04198	2.78%	[27]
12	23.754	7-Methylxanthine	166.047	7.99%	[28]

Identification of the phytochemical constituents was done using LC-ESI-MS.

## Data Availability

The data used to support the findings of this study are available from the corresponding author upon request.

## Abbreviations

AZA: Azathioprine
COX-2: Cyclooxygenase-2
DSS: Dextran sulfate sodium
EFH: Extracts of *Heritiera littoralis* fruit
LC-ESI-MS: Liquid chromatography-electrospray ionization mass spectrometry
iNOS: Inducible nitric oxide synthase
IFN-*γ*: Interferon-*γ*
IL-1*β*: Interleukin-1*β*
IL-4: Interleukin-4
IL-12: Interleukin-12
IL-17: Interleukin-17
MPO: Myeloperoxidase
NF-*κ*B: Nuclear factor-kappa B
UC: Ulcerative colitis
SASP: Sulfasalazine
TNF-*α*: Tumor necrosis factor-*α*.
